# Brigatinib treatment in a patient with advanced NSCLC with XPO1-ALK fusion: a case report

**DOI:** 10.3389/fonc.2024.1503262

**Published:** 2025-01-22

**Authors:** Yang Zhang, Ke-jie Li, Can Wang, Chang-lin Zou, Meng Su

**Affiliations:** Department of Radiotherapy, the First Affiliated Hospital of Wenzhou Medical University, Zhejiang, Wenzhou, China

**Keywords:** XPO1-ALK, brigatinib, lung adenocarcinoma, targeted therapy, case report

## Abstract

Patients with ALK-rearranged non-small cell lung cancer (NSCLC) who are treated with ALK tyrosine kinase inhibitors (ALK TKIs) have better prognoses. In this case report, we provide evidence of a novel ALK fusion, XPO1-ALK (intergenic), identified by next-generation DNA sequencing in a patient with advanced lung cancer. After 5 months of brigatinib targeted therapy, the patient clearly experienced tumor disintegration, and this treatment resulted in partial remission. To date, this patient has experienced 5 months of progression-free survival after brigatinib treatment. In addition to reporting the identification of a novel ALK fusion, XPO1-ALK (intergenic), and the sensitivity and safety of brigatinib treatment for lung cancer, this study increased the list of known ALK fusion partners in ALK-positive NSCLC. This case report has a significant clinical reference.

## 1 Introduction

NSCLC, which is a malignant tumor of epithelial origin, accounts for 85%-88% of all lung cancer cases; NSCLC includes squamous cell carcinoma, adenocarcinoma, and large cell lung cancer (1). According to the 2023 National Comprehensive Cancer Network (NCCN) guidelines (2), for patients with multiple metastases, it is recommended to first identify the pathological type. PD-L1 testing and detection of EGFR mutations, ALK, KRAS, ROS1, BRAF, NTRK1/2/3, METex14 skipping, and RET should be performed in patients with adenocarcinoma, large cell lung cancer and NSCLC not otherwise specified (NOS). If necessary, repeated tests can be performed, even when detecting a wider range of molecules. PD-L1 should be detected in patients with squamous cell carcinoma, and detection of the aforementioned genes can also be considered. The aforementioned molecular tests yield positive results for patients with numerous metastases and PS scores ranging from 0-2, allowing for the selection of targeted medications or immune preparations. For individuals with adenocarcinoma, large cell carcinoma and NOS for whom the rate of PD-L1 positivity is ≥ 1% and in whom no other gene mutations are present, carboplatin or cisplatin as well as pemetrexed and pembrolizumab are recommended treatments. For squamous cell carcinoma, the recommended drugs are carboplatin, paclitaxel or albumin-bound paclitaxel plus pembrolizumab. In patients with a rate of PD-L1 positivity less than 1%, these therapy options may also be employed.

Approximately 2%–7% of NSCLC patients have ALK gene rearrangements, which is important for treatment planning (3). The echinoderm microtubule-associated protein-like 4-anaplastic lymphoma kinase (EML4-ALK) fusion gene is the most common fusion gene that has been discovered in NSCLC thus far. The EML4 and ALK genes were found on p21 and p23 of human chromosome 2, and the orientation of the sequences of these two genes is flipped (4). The EML4-ALK fusion protein can directly form an ALK dimer without an exogenous ligand, activating ALK and the downstream RAS/ERK/STAT3/mTOR pathway and other signaling pathways (5). Ultimately, this results in the development of NSCLC.

Since second-generation ALK inhibitors, such as aletinib, brigatinib, and ensartinib, can increase progression free survival (PFS) compared to crizotinib, these latest-generation ALK inhibitors are suggested as first-line treatments for patients with ALK-positive NSCLC. However, because there are multiple uncommon fusion targets, it is critical to determine possible alternative therapeutic approaches and evaluate the clinical efficacy of ALK-TKIs when used as first-line therapies, or even posterior-line therapies, to treat patients with these recently identified fusion variants. In this case, the XPO1-ALK fusion was discovered for the first time, and the effectiveness of bugatinib was demonstrated.

## 2 Case presentation

A 51-year-old woman who did not smoke and who had no family history of genetic diseases, family history of tumors, or psychological history was diagnosed with stage IV lung adenocarcinoma in the left lower lobe and left supraclavicular lymph node, pancreas, bladder, and bone metastasis in February 2021. On presentation, a cystic space-occupying lesion was initially identified, and the patient underwent transurethral resection. Then, she underwent a PET/CT examination, and lung-occupying lesions were discovered. In addition to the mediastinal mass that was located in the basal segment of the left lower lobe and exhibited elevated metabolic activity, the reporter also detected numerous lymph nodes and multiple bone lesions that exhibited high metabolic activity throughout the body as well as nodules with high metabolic activity in the body of the pancreas and in both adrenal glands. Additionally, tumor cells were detected in the pleural effusion. The diagnosis of a primary malignant lung tumor is taken into consideration first based on the findings of the examination mentioned above. Tuberculosis infection was ruled out by pathogenic investigation, however benign tumors such as inflammatory pseudotumors must still be differently identified by pathologic biopsies. The pathological findings of lung puncture specimens confirmed that the tumor was metastatic adenocarcinoma originating from the lung, and the tumor proportion score (TPS) of PD-L1 expression was 30%. Immunohistochemical analysis revealed that TTF-1 and CK7 were positively expressed in the lung tumor puncture sample. In contrast, CK20, Syn, and gata3 were not expressed, and no other molecular mutations were found. Consequently, the patient was diagnosed with left lung adenocarcinoma with bladder and bone metastasis (cT2aN3M1c, stage IVb).

Based on these results, the patient was started on first-line carboplatin at a dose of 550 mg, pemetrexed at a dose of 700 mg and keytruda at a dose of 200 mg once every 3 weeks in March 2021. Disease re-evaluation after the first four cycles of chemotherapy combined with immunotherapy revealed an unstable response, with multiple persistent lesions in the bilateral frontoparietal temporal lobe, left occipital lobe and right cerebellum on MRI. Subsequently, her global cerebral was subjected to external radiotherapy at a dose of 30 Gy/10 fx. The patient’s initial six-course treatment plan ended in August 2021, at which point, treatment was changed to pemetrexed at a dose of 700 mg and keytruda at a dose of 200 mg once every 3 weeks until June 11, 2022. Afterward, the disease further progressed, with worse results of chest CT scan of the increasingly larger left lung lobe next to the mediastinal lesions, and the treatment plan changed again to Keytruda at a dose of 200 mg once every 3 weeks and anrotinib at a dose of 10 mg per day. Unfortunately, after four months, the regimen was changed to paclitaxel at a dose of 400 mg and keytruda at a dose of 200 mg, and the original lesion continued to worsen, as evidenced by CT scan the following month. In December 2022, the patient underwent a biopsy of the lung lesion for sequencing, and no clinically significant mutations were detected. From then until August 2023, the patient’s treatment plan was constantly changing. One cycle of targeted therapy with docetaxel and bevacizumab, two cycles with pemetrexed and Keytruda, five cycles with paclitaxel and bevacizumab, one cycle with paclitaxel and bevacizumab and Keytruda and two cycles with sintilizumab and anlotinib were used. It is no exaggeration to say that the patient’s previous chemotherapy and targeted therapy had no significant effect. At this time, the patient was reexamined by CT, and it was found that the abdominal and peritoneal lymph nodes were enlarged; this was confirmed to indicate lung adenocarcinoma metastasis, and the multiple masses in both the kidneys and the pancreas were larger than before. In October 2023, the patient suffered from persistent and unbearable pain in the epigastrium for more than a month. Considering that this pain may have been related to retroperitoneal lymph node metastasis, we administered radiotherapy at 30 Gy/10 fx. Upon physical examination, a solid lump of approximately 1 cm in diameter was felt on the patient’s left abdomen wall; nonetheless, it could not be ruled out that it was a tumor metastasis. Given the patient’s middle-aged age and the speed at which the illness progressed while receiving chemotherapy, a genetic mutation was most likely present. The patient consented to a biopsy of the tumor in the abdomen wall for DNA sequencing at the attending physician’s recommendation. The patient had a very high tumor burden before genetic testing by next-generation DNA sequencing was performed; for example, the performance status (PS) was three points, the hemoglobin level was only 65 g/L, and tumor markers such as CA153, CA125, and CA199 were highly elevated. On November 2023, the XPO1-ALK fusion gene was found to be mutated based on Next-generation Sequencing (NGS) findings, which has never been reported before. As per the guidelines for CARE Case Reports, we opted for a pharmacologic treatment approach and administered 180 mg of brigatinib once day in the hopes of controlling the patient’s illness with targeted therapy. It was an exploratory endeavor after all conventional therapies failed, and neither its safety nor effectiveness are known. We thoroughly disclosed to the patient the potential negative effects, including liver and renal damage, before to therapy. The patient accepted the treatment plan after being made aware of its hazards.

**Figure 1:**
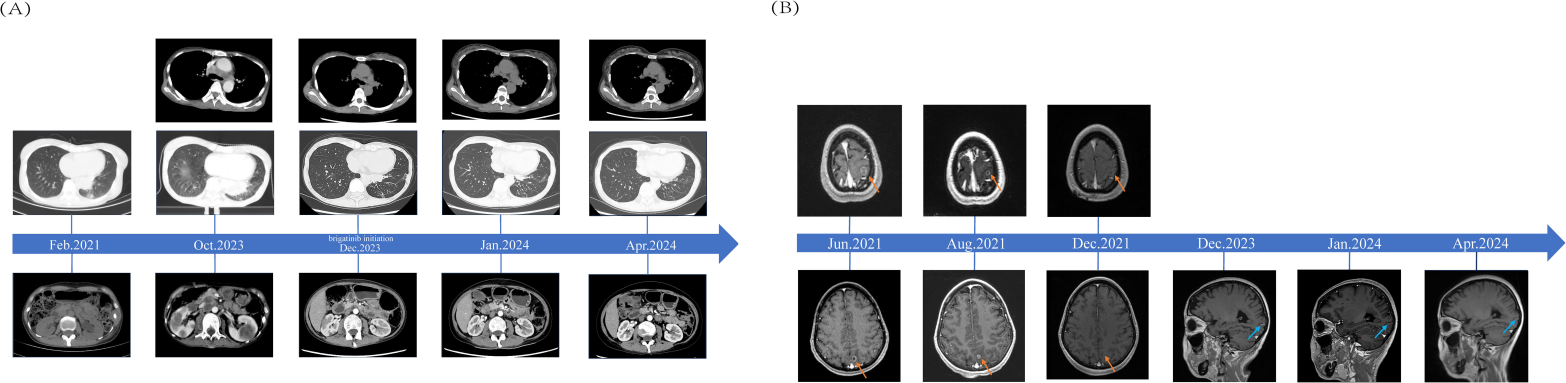
Comparisons of Patients’ Pre- and Post-treatment: **(A)** CT scans from the time when lung malignancy was discovered to 4 months after brigatinib targeted therapy and imaging data from October 2023 were used as baseline assessments. **(B)** The red arrow indicates the bilateral fronto-parieto-temporal lobes and left occipital lobe with multiple metastases in the patient’s brain before and after radiotherapy. The blue arrow indicates patchy enhancement in the left occipital cortex.

**Figure 2:**
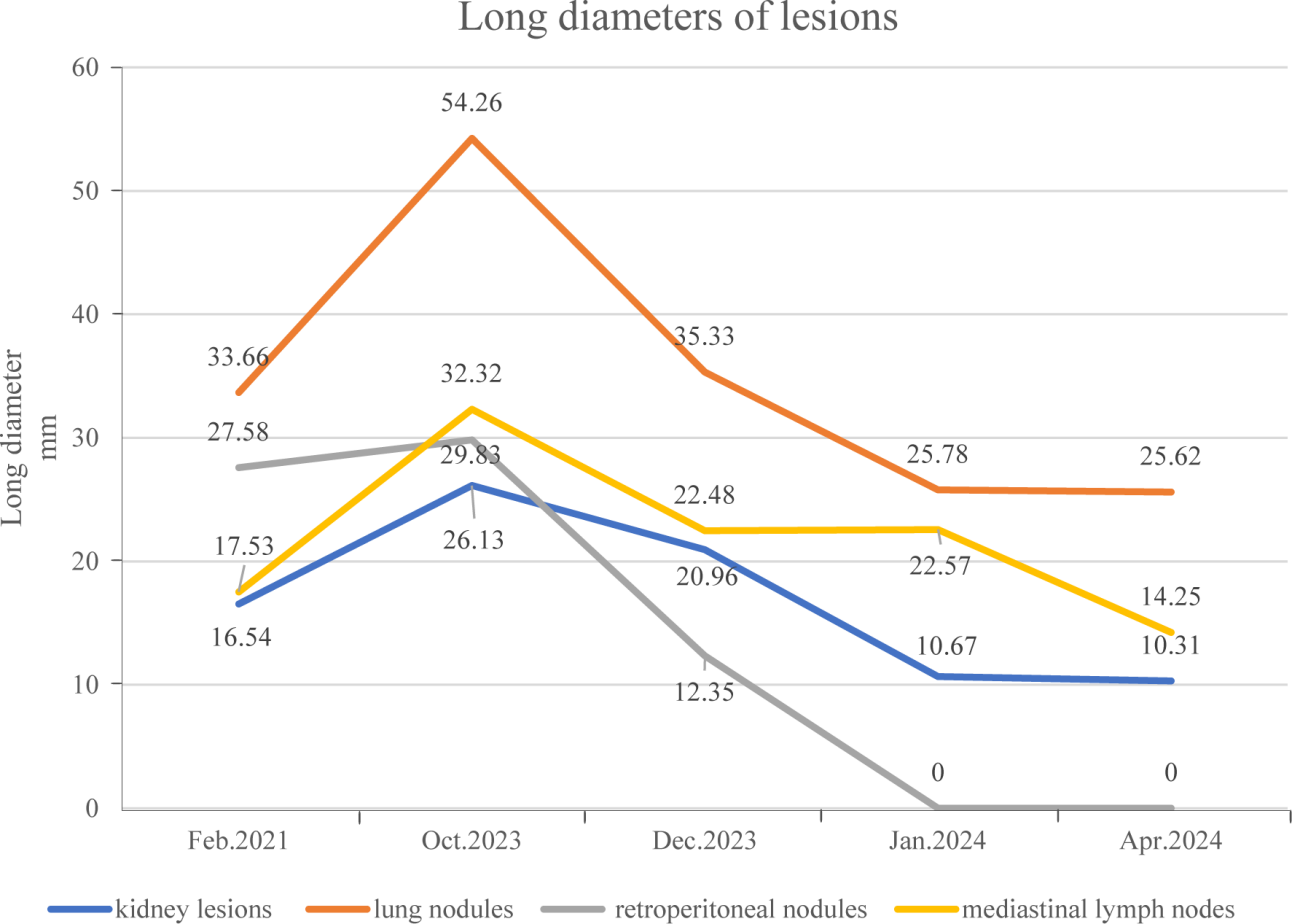
The long diameter of the patient’s pulmonary nodules and multiple metastatic lesions before and after targeted therapy.

**Figure 3:**
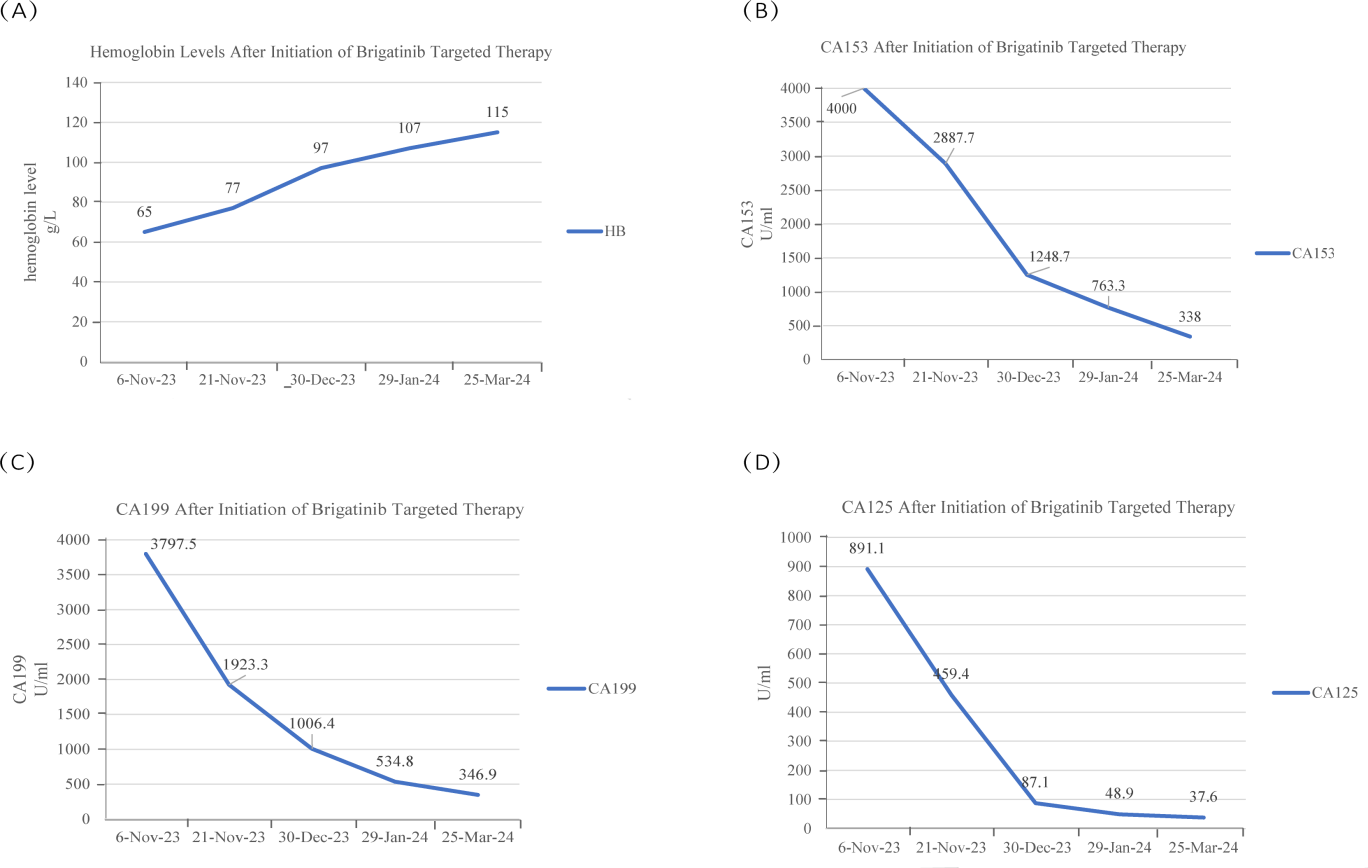
**(A)** The patient’s hemoglobin content gradually increased during brigatinib targeted therapy. **(B-D)** The CA153 concentration was greater than 4000 U/ml at the first two measurements, the CA199 and CA125 concentration peaked at the time of examination in early November, and the levels of these two tumor markers decreased after brigatinib targeted therapy.

To date, the patient has received brigatinib targeted therapy for 6 months. The response to brigatinib was a partial response (PR). Before starting brigatinib targeted therapy, we conducted a baseline assessment of the patient’s latest tumor progression data and collected all of the patient’s head, chest, and abdomen CT scans, tumor marker results, routine blood test results, and blood biochemical indicator results. In January 2024, we again evaluated the patient’s abovementioned indicators and cranial magnetic resonance. The patient in this case report had a good clinical response to brigatinib monotherapy. Transaminase elevation during treatment was the only secondary adverse reaction. As of April 2024, the patient had been taking brigatinib at a regular dose of 180 mg per day for 6 months. The patient got monthly outpatient follow-up visits from attending doctor and shown a good level of adherence and tolerance to the intervention. According to imaging examination, the primary chest lesion shrank from 35x15 mm to 25x10 mm. The paramediastinal lesions in the left lower lobe of the lung, mediastinal lymph nodes, metastatic lesions in both kidneys, and multiple retroperitoneal nodules were significantly smaller after treatment than before, and brain metastasis did not progress further. A more thorough line graph of imaging alterations and tumor size before and after therapy is displayed in **Figure 1** and **Figure 2**. The patient’s pernicious anemia improved without blood transfusions or the use of drugs to stimulate red blood cell proliferation. The levels of tumor markers, such as CA153, CA199 and CA125, also gradually decreased (6). The prognosis of lung cancer may be evaluated by tumor markers such as CA125, CA153, and CA199. CA125 is involved in cell-cell interactions that enable tumor cells to metastasize, promoting cell motility and possibly invasion (7). The CA153 level and positive rate are considerably higher in patients with distant metastases. Variations in tumor growth, local lymph nodes, and distant metastases are somewhat consistent with variations in blood CA153 levels (8). Furthermore, normal glandular epithelial cells with secretory functions contain a large amount of CA199. CA199 will enter the bloodstream as a result of tumor cell growth and necrosis, as well as the destruction and infiltration of new blood vessels (9). The patient’s general health status was re-evaluated, and the PS score was 0. **Figure 3** presents a few of the aforementioned statistics visually. All the procedures that were performed in this study were performed in accordance with the ethical standards of the institutional and/or national research committee and with the Declaration of Helsinki (2013 revision). Written informed consent was obtained from the patient for publication of this case report and imaging data.

## 3 Discussion

The XPO1-ALK fusion is a rare alteration that was first described in the present report; additionally, this report describes the subsequent follow-up of a patient with NSCLC harboring the XPO1-ALK fusion.

Among ALK fusion mutations that occur in NSCLC, EML4 is the most common gene fusion partner in ALK-rearranged lung adenocarcinoma. Some studies have explored the comparative efficacy of therapeutic drugs as well as the mechanisms underlying drug resistance in NSCLC patients harboring the EML4-ALK fusion (10). In addition to EML4-ALK, the more common types of ALK gene fusion mutations include HIP1-ALK and STRN-ALK (11). Other rare fusion genes that were discovered in recent years include the LOC399815-ALK fusion (12), sperm antigen with calponin homology and coiled-coil domains 1-like (SPECC1L)-ALK fusion (13), ring finger protein 144A (RNF144A)-ALK fusion (14), kinesin light chain 1 (KLC1)-ALK fusion (15), SLC34A2-ALK fusion (16), carboxypeptidase E (CPE)-ALK fusion (17), LIM zinc finger domain containing 1 (LIMS1)-ALK fusion (18), SET domain containing 3 (SETD3)-ALK fusion (19), LOC388942-ALK fusion (20), human immunodeficiency virus type I enhancer binding protein 1 (HIVEP1)-ALK fusion (21), and SEC31A-ALK fusion (22). **Table 1** provides a more thorough explanation of ALK fusion type discoveries and their therapeutic implications. Among the many genes that have been shown to be rearranged with ALK, there are currently no reports on the XPO1-ALK fusion in metastatic NSCLC, and there are no clear conclusions on whether using ALK-TKIs is effective or safe.

**Table 1:** Summary of treatment efficacy in Rare ALK-fusion-positive NSCLC.

Reference	Rare ALK fusion types	ALK-TKIs	Response
Gu, X. et al. (21)	HIVEP1-ALK	Alectinib	PR > 33 m
Zhai, X. et al. (20)	LOC388942-ALK	Crizotinib, Ceritinib, Aletinib	OS > 5 y
Dai, S. et al. (19)	SETD3-ALK	Crizotinib	PFS > 16 m
Kim, R. N. et al. (22)	SEC31A-ALK	Crizotinib	PR > 12 m
Shi, J. et al. (18)	ALK-LIMS1	Crizotinib	PFS: 2 m
Qin, Y. et al. (17)	CPE-ALK	Alectinib	PFS > 5 m
Siblini, L. et al. (15)	KLC1-ALK	Alectinib, Brigatinb and Lorlatinib	PFS: 6 w PFS: 5 w PFS: 7 d
Zhang, Q. et al. (13)	SPECC1L-ALK	Iruplinalkib	PFS: 39.3 m
Feng, T. et al. (23)	TNIP2-ALK	Crizotinib	PFS > 10 m
Li, L. et al. (24)	TFG-ALK	Crizotinib	PFS = 3 m
Cao Q, et al. (25)	NCOA1–ALK	Crizotinib	PFS > 11 m
Fang, W. et al. (26)	MPRIP-ALK	Alectinib, Ensartinib	PD PFS > 18 m
Li, M. et al. (27)	LMO7-ALK	Crizotinib	PFS > 6 m
Chen, H.F. et al. (28)	SOS1-ALK	Crizotinib	PFS: 23 m
Tian, P. et al. (29)	CENPA-ALK	Crizotinib	PFS: 15 m
Tian, P. et al. (29)	TACR1-ALK	Crizotinib	PFS: 7 m
Tian, P. et al. (29)	LOC349160-ALK	Crizotinib	PFS: 18 m
Tian, P. et al. (29)	CHRNA7-ALK	Crizotinib Ceritinib	PFS: 11 m PFS > 9 m
Zeng, H. et al. (30)	KIF5B-ALK	Crizotinib Ceritinib	PFS: 11 m PFS > 9 m
Tian, P. et al. (29)	DYSF-ALK ITGAV-ALK	Crizotinib	PFS: 15 m
Li, H. et al. (14)	ALK-RNF144A; HIP1-ALK fusion	Crizotinib	PFS > 18 m
Li, Y. et al. (12)	LOC399815-ALK; ALK-EML4	Alectinib	PFS > 29 m
Wu, X. et al. (31)	CCNY-ALK; ATIC-ALK	Crizotinib	PFS > 10 m
Wu, X. et al. (32)	NLRC4-ALK; EML4-ALK	Crizotinib Ceritinib	PFS: 6 m PFS > 2 m
Luo, J. et al. (33)	PRKCB-ALK; EML4-ALK	Crizotinib	PFS: 13 m
Qin, B. et al. (34)	BCL11A-ALK; EML4-ALK	Crizotinib	PFS > 11 m
Lin, H. et al. (35)	FBXO11-ALK; EML6-ALK	Crizotinib	PFS > 3 m
Yin, J. et al. (36)	DYSF-ALK; ITGAV-ALK	Crizotinib	PFS: 9 m
Tao, H. et al. (37)	ALK-SSH2; EML4-ALK	Crizotinib	PFS: 12 m
Tao, H. et al. (37)	ARID2-ALK; EML4-ALK	Crizotinib	PFS: 23 m
Guo, J. et al. (38)	CDK15-ALK; EML4-ALK	Alectinib	PFS > 11 m
Zeng, H. et al. (39)	PDK1-ALK; STRN-ALK	Alectinib	PFS > 20 m
Zhai, X. et al. (40)	ALK-GCA; EML4-ALK	Alectinib	DFS: 20 m

Brigatinib, which is a type of second-generation ALK-TKI, can inhibit ALK fusion and mutated EGFR (L858R) and ROS1 fusion, and it has been demonstrated to exert better therapeutic effects than first-generation ALK-TKIs in previous clinical studies. For example, in a *post hoc* analysis of a phase 3 study titled “Anaplastic Lymphoma Kinase in Lung Cancer Trial of Brigatinib in the First Line (ALTA-1L)”, the PFS rate was 24 months in the brigatinib group and 11 months in the crizotinib group (HR=0.49; 95% CI 0.33−0.74) (41). In addition, the ORRs in the brigatinib group and crizotinib group were 71% and 60%, respectively (42). In addition, because brigatinib exhibits a superior ability to penetrate the blood-brain barrier, it can also achieve better control of intracranial metastases. The results suggested an overall survival benefit when brigatinib was used to treat patients with baseline brain metastases (HR = 0.43, 95% CI: 0.21–0.89) (43). This study also showed that newly emerged secondary ALK mutations are rare in patients who progress on brigatinib targeted therapy. This finding suggested that brigatinib inhibits mutations that are associated with resistance to ALK-TKIs.

In the pooled 13 randomized controlled trials, there was no significant difference between brigatinib and other ALK-TKIs in terms of adverse events (AEs). The incidence rate of grade 3-4 AEs related to brigatinib was 63.7%, and the most frequent AEs were gastrointestinal reactions, hypertension, cough, headache, and elevated ALT or AST levels (44). According to the safety exposure-response analysis of the phase I/II and phase II ALTA studies, grade ≥ 2 rash and amylase tended to occur more frequently with increasing brigatinib exposure (45). There were 136 patients who received brigatinib 180 mg once daily in the ALTA-1L study; 12 of them discontinued treatment due to adverse reactions, and 29 patients experienced dose reduction (41).

Herein, we report a NSCLC patient with the XPO1-ALK gene fusion for whom two previous NGS tests did not yield valuable findings. The patient received pemetrexed, carboplatin and immunotherapy. After many rounds of progression and changes in the 9-line treatment regimen, brigatinib monotherapy was chosen as the 10th line of treatment. Ultimately, the primary tumor and metastases were effectively controlled with no extreme drug-related adverse reactions. This unique case report not only revealed an unprecedented new ALK fusion gene but also indicated that XPO1-ALK was sensitive to brigatinib. However, due to the limited follow-up time, we are still unable to determine the time when resistance to brigatinib occurs. In addition, since the XPO1-ALK fusion mutation was detected only in the NGS of one patient, it is difficult to confirm whether other patients with the same mutation are also highly sensitive to brigatinib targeted therapy; addressing this question will require the identification of more patients harboring the XPO1-ALK fusion in the future.

## 4 Conclusion

In summary, among the current reports about gene mutations in NSCLC, no XPO1-ALK gene fusion mutations have been identified. This case involved the discovery of the XPO1-ALK fusion and demonstrated that a patient with multiple organ metastases and lung adenocarcinoma harboring an XPO1-ALK fusion mutation responded effectively to brigatinib targeted therapy without serious adverse effects that could cause dosing changes.

## Data Availability

The datasets presented in this article are not readily available because of ethical and privacy restrictions. Requests to access the datasets should be directed to the corresponding author.
